# Supplementary material for: Association between the use of Information and Communication Technology and activeness in life among community-dwelling older adults
Source: PLoS One. 2024 Nov 20;19(11):e0311139. doi: 10.1371/journal.pone.0311139 (PMC11578520; doi:10.1371/journal.pone.0311139)
Supplement: Attachment — Submitted filename: Response to Reviewers.docx [file pone.0311139.s002.docx]

Dr. Tadashi Ito

Academic Editor

*PLOS ONE*

Dear Dr. Tadashi Ito:

We wish to re-submit the manuscript titled “Association between the use of information and communication technology and activeness in life among community-dwelling older adults.” The manuscript ID is PONE-D-24-14960.

We thank you and the reviewers for your thoughtful suggestions and insights. The manuscript has benefited from these insightful suggestions. I look forward to working with you and the reviewers to move this manuscript closer to publication in the journal *PLOS ONE*.

The manuscript has been rechecked and the necessary changes have been made in accordance with the reviewers’ suggestions. The responses to all comments have been prepared and are presented below.

Thank you for your consideration of the revised paper.

Sincerely yours,

Kanna Tezuka, RN, MSN

Graduate School of Nursing, Osaka Metropolitan University

Osaka, Japan

Email: so22611b@st.omu.ac.jp

PONE-D-24-14960

“Association between the use of Information and Communications Technology and activeness in life among community-dwelling older adults”

***Reviewer＃１***

1. ***As shown, the questionnaire was distributed to 4,793 participants, only 1,057 responded***

***(response rate of 22.1%), which might cause selection bias and other biases, as those who responded might have better activeness.***

As the reviewer pointed out, the participants in this study engagement in activities may be relatively higher, possibly leading to selection bias. Nonetheless, this study clearly showed an association of ICT use with a high level of activeness in life (expansion of life space and motivation). Therefore, we have added a comment regarding the possibility of these biases as a study limitation in the revised manuscript (page 21, lines 334-336).

***２．The paper was poorly written and hard to understand. English expressions should be further refined.***

　Addressing the reviewer’s comment, we have extensively revised the manuscript and have attempted to ensure that the rewritten manuscript is clear and comprehensible. The manuscript has also been checked by an English proofreading service and the proofreading certificate has been attached for reference.

***３．Table2 and 3 could be combined. And the expression of tables in the text (such as line 206-207) is not appropriate.***

According to the reviewer’s comment, we have combined Table 2 and Table 3 in the original manuscript into Table 2 in the revised paper. In addition, we have inserted Tables 1 and 2 immediately after the first paragraph in which they are cited according to the submission guidelines and confirmed that the tables are accurately formatted with appropriate captions in the revised manuscript.

PONE-D-24-14960

“Association between the use of Information and Communications Technology and activeness in life among community-dwelling older adults”

***Reviewer＃2***

***It is believed that this report was written with careful consideration of demographic characteristics to suit the topic. I think this is a very well written report. However, it is judged that the statistical aspect will require advice from experts in other statistical fields.***

According to the reviewer’s comment, we have asked an expert in statistics, Professor Kazuya Ito, Statistician, Graduate School of Nursing, Osaka Metropolitan University, to evaluate the statistical analysis conducted in this study. Consequently, the statistical analysis procedures were critically reviewed and validated by him. We have added a comment regarding this in the revised manuscript (page 22, lines 356-358) and attached the certificate of statistical analysis.
